# Operations on the Liver

**Published:** 1898-03-19

**Authors:** Leonard A. Bidwell

**Affiliations:** Senior Assistant Surgeon to the Hospital


### Medical Progress and Hospital Clinics.

[*The Editor will be glad to receive offers of co-operation and contributions from members of the profession. All letters should be addressed to* The Editor, at the Office, 28 & 29, Southampton Street, Strand, London, W.C.]

### OPERATIONS ON THE LIVER.

A Postgraduate Lecture delivered at the West London Hospital by Leonard A. Bidwell, F.R.C.S., Senior Assistant Surgeon to the Hospital.

Systematic operations on the liver, except the incision of a hepatic abscess, have practically only been undertaken during the last 20 or 25 years, and during the first half of this time the results were anything but satisfactory; now, however, thanks principally to the more rational treatment of peritoneal wounds, the mortality of operations on the liver is extremely small, and these operations may be claimed to be some of the most successful in surgery.

The various diseases of the liver in which operation may be required are: (1) Abscess, which may be due to (*a*) injury, (*b*) gall-stones, (*c*) suppurating hydatid cyst, (*d*) tubercle, (*e*) actinomycosis, (*f*) pyæmia, and (*g*) the so-called tropical abscess usually dependent on dysentery; (2) hydatid cyst; (3) injuries, *i.e.*, penetrating wound or rupture; (4) displacement of the liver; (5) tumours; (6) gall-stones; and (7) biliary fistula.

In abscess of the liver the principal symptoms are enlargement of the organ with bulging in one direction, increase of the liver dulness, increase in the width of the intercostal spaces, high temperature, and constitutional disturbance. The operation is called hepatotomy, and should be performed in two stages. An incision either transverse or vertical is made through the anterior abdominal wall over the most prominent part of the swelling down to the liver; if adhesions have formed between the parietal peritoneum and the liver, the abscess can be opened at once; if not, it is best to plug the wound with gauze, and to wait two or three days until adhesions have formed, before incising the liver. The second stage can be performed without any anæsthetic, since the incision of the liver does not give rise to any pain. An aspirator is plunged into the abscess cavity, and a long director is then passed into the cavity through the canula of the aspirator, which is then withdrawn; a long, straight bistouri is passed along the groove in the director, and a free incision made; a large drainage-tube is introduced, and the cavity washed out with a dilute antiseptic solution. There need be no fear of hæmorrhage on incising the liver with a knife, since the surrounding tissue has become condensed by the inflammation, and so doe not bleed. If any bleeding should occur, it can readily be controlled by inserting a small gauze plug by the side of the drainage-tube. Several French surgeons urge that the liver should be incised with a thermo-cautery, but this is quite unnecessary. Recently it has been recommended that the operation should be performed in one stage, but in such cases there is a great risk of the escape of pus into the peritoneal cavity, and so the procedure is undesirable.

I wil briefly relate two typical cases of abscess of the liver in which the operation was performed in the way I have described.

Case I.: A gentleman, aged 32, contracted dysentery and abscess of the liver in Java, and was sent home for operation in July, 1890. An incision was made one finger's-breadth below and parallel to the costal arch, and the surface of the liver exposed, the wound was plugged, and the abscess opened on the third day. The drainage-tube was removed on the eleventh day, and the wound was healed a week later, and the patient got up. The dysentery, however, recommenced, and the patient died some weeks later from exhaustion.

Case II.: J. G., aged 42, was admitted into the West London Hospital, under the care of Dr. Ball, on July 19th, 1892. While in India, 14 years previously, he had an attack of dysentery. He left India 10 years before admission. The present illness had commenced six weeks before. The liver dulness was increased, there was bulging of the intercostal spaces, both in front and behind, and pain just above the umbilicus. The temperature was raised. On August 2nd an incision, four inches long, was made parallel to the costal arch, and the surface of the liver exposed and found to be free from any adhesions; the parietal peritoneum was stitched to the skin, and the wound packed with gauze. On August 5th the plug was removed, and the abscess opened with a bistouri evacuating one pint of pus. The drainage tube was removed on the tenth day, and the wound was healed by August 29th. The recovery was perfect.

If the abscess is situated in the posterior part of the liver, it is best reached by an incision over the posterior portion of the ninth rib; this rib is resected, and the abscess reached through both layers of the pleura and the diaphragm.

The following case will illustrate this method of operating:—

Case III.: A gentleman, aged 30, contracted abscess of the liver in the West Indies, and was sent home for operation. There was considerable enlargement of the liver, with bulging of the intercostal space, and a hectic temperature. A hypodermic needle discovered pus in the posterior part of the liver, so an incision was made over the posterior part of the ninth rib and portion of the rib resected; on opening the pleural cavity, contrary to the teaching of physiology books, there was no collapse of the lung (it even had to be pushed out of the way); an incision was then made in the diaphragmatic pleura, and this and the costal layers of pleura were united with some points of silk suture, shutting off the pleural cavity; the diaphragm was then divided and the liver exposed. As firm adhesions were found the abscess was opened at once, and a large quantity of pus evacuated. The patient made a good recovery, and did not suffer from any pleuritic trouble.

**Hydatid Cyst.**—In hydatid disease there is a painless enlargement of the liver or a part of it without any other symptoms, and an exploring needle yields a little clear fluid containing no albumen and a little salt. The operation of hepatotomy can be performed in one stage for these cases, since as the hydatid fluid is not purulent there is not the same risk of fouling the peritoneum if any enter it when the cyst is opened. After the daughter cysts have been cleared out as fully as possible, the cyst wall should be sutured to the parietal peritoneum, and the cavity packed with gauze. The prognosis is very good.

*(To be continued.)*

